# Screening for hyperglycaemia in pregnancy and pregnancy outcomes among Aboriginal women in remote communities of the Northern Territory, Australia: a retrospective cohort study

**DOI:** 10.1136/bmjopen-2025-110242

**Published:** 2026-05-19

**Authors:** Anna J Wood, Chloe O’Hara, Ariella Joyce-Tubb, Vanya Webster, Mary Wicks, Paula Van Dokkum, Louise J Maple-Brown, Matthew J L Hare

**Affiliations:** 1 Menzies School of Health Research, Charles Darwin University, Darwin, Northern Territory, Australia; 2 Department of Endocrinology, Royal Darwin Hospital, Darwin, Northern Territory, Australia; 3 Flinders University, Darwin, Northern Territory, Australia; 4 Department of Medicine, Alice Springs Hospital, Alice Springs, Northern Territory, Australia; 5 Department of Obstetrics and Gynaecology, Alice Springs Hospital, Alice Springs, Northern Territory, Australia

**Keywords:** EPIDEMIOLOGY, Diabetes in pregnancy, Australian Aboriginal and Torres Strait Islander Peoples, Diabetes & endocrinology

## Abstract

**Abstract:**

**Background:**

Aboriginal women in the remote Northern Territory (NT) experience high rates of adverse pregnancy outcomes related to hyperglycaemia in pregnancy. Oral glucose tolerance test (OGTT) screening was recommended in early pregnancy but barriers to uptake exist.

**Objectives:**

To examine uptake of screening for hyperglycaemia in pregnancy among Aboriginal women in remote NT communities and explore adverse pregnancy outcome rates among women who did not have early OGTT screening compared with women who did undergo screening in early pregnancy and those with pre-existing diabetes.

**Design:**

Retrospective observational cohort study of pregnancies among Aboriginal women in remote NT clinics from January 2017 to December 2019. Screening for hyperglycaemia in pregnancy included having an early OGTT (<20 weeks of gestation) or a routine OGTT (≥20 weeks). Logistic regression was used to assess adverse pregnancy outcomes between those who did and did not have early OGTT screening and those with pre-existing diabetes.

**Results:**

Among 1191 pregnancies in 52 remote communities, pre-existing type 2 diabetes (T2D) was diagnosed in 6.4% (n=76) and gestational diabetes mellitus (GDM) was diagnosed in 13% (154/1191). Excluding women with pre-existing diabetes, 226 (20%) had an early OGTT. Guideline-directed screening (with either (a) an early OGTT diagnosing GDM or (b) a negative early OGTT followed by a routine OGTT) occurred in 14% of pregnancies (n=158). Compared with women who had an early pregnancy OGTT, the combined adverse pregnancy outcome was more common among women with pre-existing T2D (89% vs 54%, adjusted OR 6.06 (95% CI 2.75 to 13.35)) and similar among women who did not undergo early OGTT (50%, adjusted OR 0.97 (95% CI 0.71 to 1.32)).

**Conclusion:**

Uptake of guideline-directed screening in Aboriginal women in remote NT was low, although there was no difference in pregnancy outcomes for women who were and were not screened with an early OGTT. Rates of adverse pregnancy outcomes were concerningly high in women with pre-existing T2D, highlighting a need to strengthen diabetes care for these women.

**STRENGTHS AND LIMITATIONS OF THIS STUDY**

- This study explores hyperglycaemia in pregnancy screening and outcomes in a priority population known to experience substantial health inequities due to diabetes.
- Detailed data collection was undertaken for all pregnancies in a large number of remote communities across a vast geographic area, including manual review of electronic and paper records to minimise the risk of missing or inaccurate data.
- Gestational diabetes mellitus screening and diagnosis guidelines were standardised across all included clinics and throughout the study period.
- Retrospective design is susceptible to unmeasured confounding, bias and lack of standardised adverse outcome ascertainment.

## Background

 Rates of hyperglycaemia in pregnancy (including gestational diabetes, overt diabetes in pregnancy and pre-existing type 2 diabetes (T2D))^1^ are high for Aboriginal and Torres Strait Islander women, with one-in-four pregnant Aboriginal women in the Northern Territory (NT) experiencing hyperglycaemia.^2^ Hyperglycaemia in pregnancy contributes to persistent inequities in maternal and neonatal pregnancy complications,^3 4^ long-term adverse health outcomes for women,^5 6^ altered childhood growth and development^7 8^ and high rates of diabetes and obesity in the next generation.^9 10^ Multiple factors are likely to be driving these pregnancy and longer-term outcomes, with social determinants of health, including poverty, discrimination and the ongoing effects of colonisation, playing a considerable role.^11^ Geographical remoteness is also an important factor^12^ harbouring a transient health workforce, who play a key role in providing early pregnancy check-ups, care and referrals as needed. Many Aboriginal women in the NT travel to larger towns, away from the support of their home and families, for some aspects of their care, particularly if they have hyperglycaemia or as they approach their due date. Aboriginal and Torres Strait Islander peoples in Australia living in remote communities are at the greatest risk of developing T2D.^13^ This is particularly concerning in the NT context, where Aboriginal people make up 30% of the population, a much higher proportion than in other states and territories, with 80% of Aboriginal people living in remote areas.^14^

In Australia, universal screening for gestational diabetes mellitus (GDM) is recommended using a 2-hour 75 g oral glucose tolerance test (OGTT) at 24–28 weeks of gestation (a routine OGTT).^15^ For women considered to be at high risk, including all Aboriginal women, an early OGTT at the first opportunity after conception was recommended during the study period, followed by a routine OGTT at 24–28 weeks of gestation if the early OGTT is consistent with normoglycaemia.^15^ The Australasian Diabetes in Pregnancy Society GDM screening recommendations have since been updated, with changes to early pregnancy screening recommendations.^16^ Local guidelines in the NT suggest a glycated haemoglobin (HbA1c) and venous blood glucose level at the first antenatal visit if an OGTT is not feasible.^17^

Importantly, early screening for hyperglycaemia in pregnancy allows for the detection of overt diabetes in pregnancy (overt DIP), defined as newly detected hyperglycaemia during pregnancy, with plasma glucose or HbA1c levels meeting criteria for T2D diagnosed outside of pregnancy.^15^ Women diagnosed with overt DIP are an important group to identify, as they are at high risk of having undiagnosed T2D or rapid progression to T2D post partum.^6^

Despite early screening with an OGTT for Aboriginal women being recommended by local and national guidelines at the time of this study, there are limited real-world data on screening rates across remote areas of the NT. Given the low uptake of early screening with an OGTT described more broadly in the literature,^18^ uptake in our context is suspected to be low, and whether alternate tests such as an HbA1c or isolated plasma glucose levels (fasting or random) are being used instead is not known.

Our study aimed to address these gaps in the literature to report on (a) rates of hyperglycaemia screening as per guidelines, as well as rates of screening with a routine OGTT only or an HbA1c or fasting/random plasma glucose and (b) pregnancy outcomes among women who did and did not have an early OGTT and those with pre-existing T2D, for Aboriginal women in remote NT. We hypothesised that rates of early pregnancy OGTT uptake would be low and that women who did not undergo screening with an early OGTT would have an elevated risk of adverse pregnancy outcomes due to limited engagement with guideline-directed care compared with those who underwent early pregnancy screening.

## Methods

### Study population and data sources

**Figure 1:**
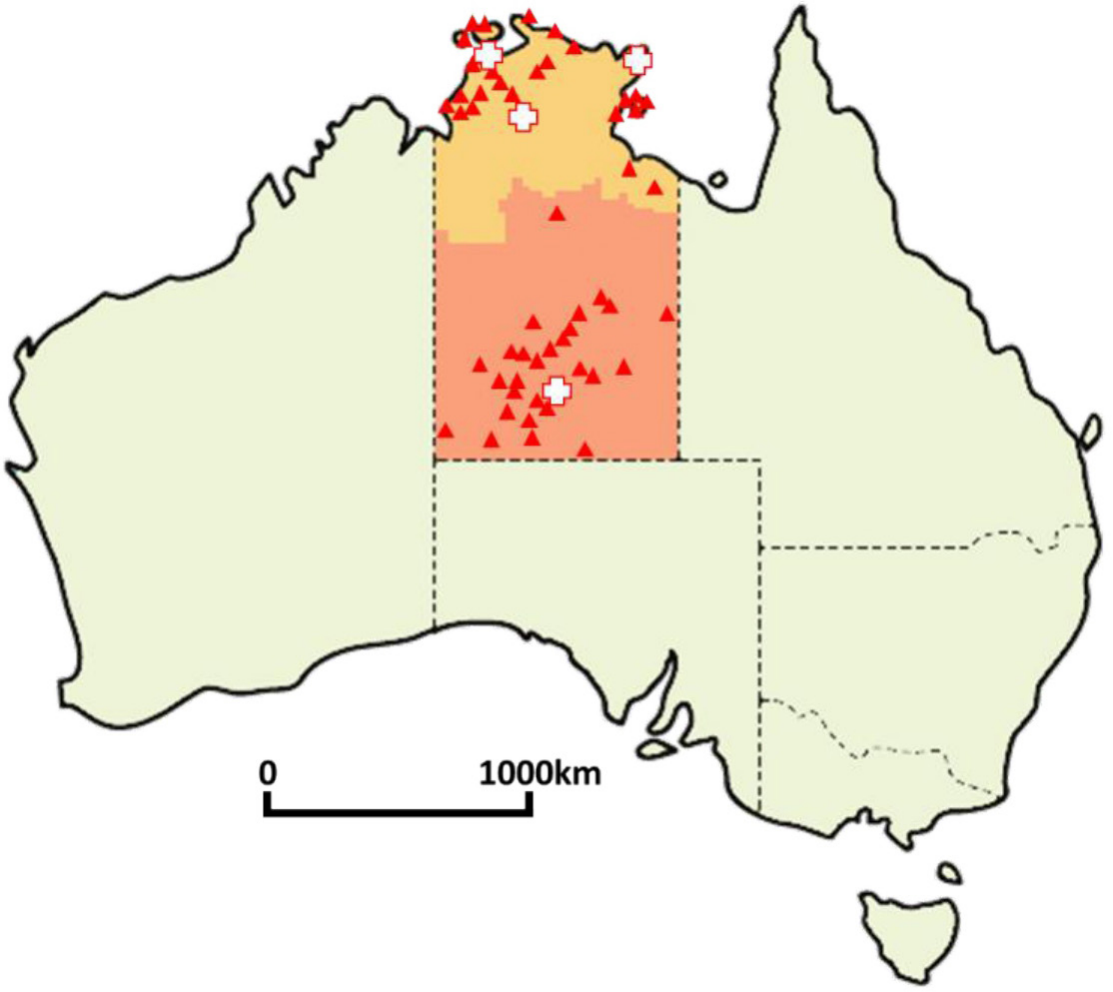
Map of Australia showing the Northern Territory and approximate locations of the 52 remote clinics and 4 public hospitals that contributed data to the study.

In the NT, remote healthcare is provided by Aboriginal community-controlled or government (NT Health) services. Each clinic is the sole health service for the local remote community. The sites for this study were all 52 remote centres managed by NT Health and all NT public hospitals with maternity units (figure 1). Our study cohort included all pregnancies among Aboriginal women with a delivery date between 1 January 2017 and 31 December 2019, as recorded in the NT Perinatal Data Collection, who received any antenatal care at an NT Health remote clinic, as indicated by the presence of an antenatal service item or antenatal care plan in the NT Health Primary Care Information System (PCIS). PCIS is the electronic medical record system used across all 52 remote clinics. The Perinatal Data Collection is a register of all births in the NT, including non-hospital births. Data are entered by clinicians immediately following a birth.

### Data collection

Data were initially extracted from the NT Health PCIS and NT Perinatal Data Collection. Extracted data were deterministically linked using a unique personal identifier, the hospital reference number, which is used universally across all NT Health services and has been validated for data linkage.^5^ Data extracted from PCIS included relevant biochemistry (HbA1c and plasma glucose levels) and anthropometry. The NT Perinatal Data Collection provided data on demographic and clinical characteristics as well as pregnancy outcomes. Where no early pregnancy OGTT results were available in extracted data, primary care and hospital electronic medical records (n=904) were manually reviewed. If it was still unclear whether or not appropriate screening had occurred, paper medical charts from the four NT public hospitals with birth units were reviewed (n=626). Manual data collection was conducted using a standardised electronic form by two medical students (CO’H, AJ-T) and two research nurses (PVD, VW).

### Screening and birth outcome definitions

An early OGTT was defined as an OGTT performed prior to 20 weeks’ gestation and a routine OGTT as an OGTT performed at 20 weeks or more of gestation. Guideline-directed screening was defined as an early OGTT diagnostic of GDM or an early normoglycaemic OGTT followed by a routine OGTT. No screening included no form of glycaemic testing (no OGTT, HbA1c, fasting/random plasma glucose) throughout pregnancy. Large for gestational age (LGA) and small for gestational age (SGA) were defined as neonatal birth weights greater than the 90th or less than the 10th centile, respectively, according to national birth weight percentiles by sex and gestational age.^19^ Caesarean section included elective and emergency procedures. Pre-eclampsia excludes isolated antenatal or perinatal hypertension. Pre-term delivery refers to delivery before 37 weeks of gestation. An admission of any duration to the neonatal intensive care unit (NICU) or special care nursery was included. The combined adverse outcome included at least one of LGA, caesarean section, pre-eclampsia, pre-term delivery or admission to NICU.

Pre-existing T2D was defined as a diagnosis of T2D recorded in the NT Primary Health Care Information System or NT Perinatal Data Collection. GDM was defined as (a) recorded in the NT Primary Health Care Collection or NT Perinatal Data Collection or (b) diagnosed using a 2-hour 75 g OGTT and defined as fasting plasma glucose level 5.1–6.9 mmol/L, 1-hour plasma glucose ≥10.0 mmol/L, 2-hour plasma glucose 8.5–11.0 mmol/L.^20^ Overt DIP was defined by any of fasting plasma glucose ≥7.0 mmol/L, 2-hour plasma glucose ≥11.1 mmol/L and/or HbA1c≥6.5%. Hyperglycaemia in pregnancy was first detected in pregnancy and included both GDM and overt DIP.

### Covariates

Maternal parity referred to the number of previous live births at the time of conception. First, antenatal body mass index (BMI) was the first recorded BMI measurement during antenatal care as recorded in the NT Perinatal Data Collection. Any smoking and alcohol use during pregnancy was included. Pre-existing hypertension, cardiac and renal disease prior to pregnancy were each defined as a diagnosis of disease prior to estimated conception for this pregnancy as recorded in the NT Primary Health Care Information System and/or NT Perinatal Data Collection.

### Statistical methods

Statistical analysis was conducted using Stata V.17 (Stata Corporation, College Station, Texas, USA). Descriptive data were presented as numbers (%), mean (SD) or median (IQR). Differences in maternal characteristics between early screening groups (did have early OGTT, did not have early OGTT) and pre-existing T2D were determined using Pearson χ^2^ tests for categorical variables and analysis of variance for continuous variables.

Clinical outcomes of singleton pregnancies were compared between women who did not have an early OGTT, women who had pre-existing T2D and women who had an early OGTT. Results are expressed as absolute numbers and percentages, with differences determined using logistic regression. ORs are presented both unadjusted and adjusted for factors with known associations to adverse pregnancy outcomes (maternal age, smoking during pregnancy (yes vs no), BMI and parity). Variables included in regression models were selected a priori based on clinical plausibility. Cluster-robust SEs were used to account for lack of independence in the sample due to a small number of women who had multiple pregnancies within the study period.

## Results

In the 3-year study period, there were 1191 pregnancies to 1141 Aboriginal women who received antenatal care in NT Health remote primary care centres. The mean maternal age was 25.1 years (95% CI 24.8 to 25.4). Of the 1191 pregnancies, 76 (6.4%) had pre-existing T2D and no women had pre-existing type 1 diabetes.

### Glycaemic screening

**Figure 2:**
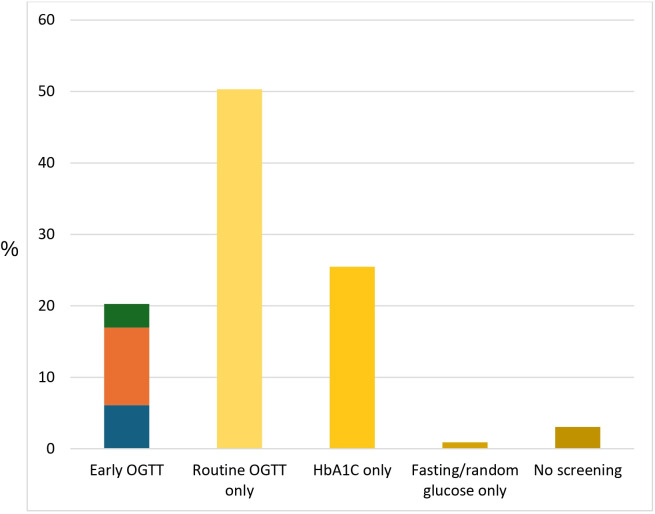
Rates of hyperglycaemia screening for Aboriginal women in remote communities in the Northern Territory between 2017 and 2019 (n=1115). Green represents participants with GDM on early OGTT; orange represents participants with negative early OGTT and subsequent routine OGTT; and blue represents participants with negative early OGTT and no subsequent routine OGTT. Early OGTT, an OGTT performed prior to 20 weeks of gestation; HbA1C, glycated haemoglobin; GDM, gestational diabetes mellitus; OGTT, 75 g oral glucose tolerance test; routine OGTT, an OGTT performed at 20 weeks or more of gestation.

Excluding pregnancies complicated by pre-existing T2D, rates of glycaemic screening are outlined in figure 2 (n=1115). Screening with an early OGTT occurred in 20% (n=226) of pregnancies, while screening as per guidelines occurred in 14% of pregnancies (n=158). There were 284 pregnancies (26%) where an HbA1c was performed without an OGTT and 34 pregnancies (3.0%) with no glycaemic screening during pregnancy (figure 2).

Hyperglycaemia in pregnancy was newly detected in 3.3% (n/N=37/1115) of all pregnancies at early screening, with 2.8% (n/N=31/1115) meeting criteria for GDM and 0.5% (n/N=6/1115) meeting criteria for overt DIP. When an early OGTT was performed, 16% (n/N=37/226) met criteria for hyperglycaemia in pregnancy. Of those who had a negative early OGTT, 36% (n/N=68/189) did not go on to complete a routine OGTT later in pregnancy as per guidelines. An early HbA1c was performed in 69% (n/N=774/1115) of all pregnancies without pre-existing diabetes. One woman had an early HbA1c≥6.5%.

A routine OGTT was undertaken in 63% (n/N=682/1078) of eligible pregnancies. Hyperglycaemia in pregnancy was detected in 11% (n/N=117/1078), with 9% (n/N=98/1078) meeting criteria for GDM and 1.3% (n/N=14/1078) meeting criteria for overt DIP. When a routine OGTT was performed, 17% (n/N=117/682) met criteria for hyperglycaemia in pregnancy.

#### Differences in baseline characteristics between those who did and did not have an early OGTT and pre-existing T2D

Women who did not have an early OGTT, compared with women who were screened with an early OGTT and women with pre-existing T2D, were younger (24 years vs 26 years vs 30 years, p<0.01, respectively), had a lower BMI (25 kg/m^2^ vs 27 kg/m^2^ vs 31 kg/m^2^, p=0.001) and had high rates of smoking (54% vs 45% vs 43%, p=0.045). Women screened with an early OGTT first presented for antenatal care earlier in gestation compared with women without an early OGTT and those with pre-existing T2D (7 weeks vs 10 vs 9 weeks, p<0.01, respectively). Rates of pre-existing medical comorbidities were lower in women with and without an early OGTT, compared with those with pre-existing T2D (9.6% vs 12% vs 32%, p<0.01). More women screened with an early OGTT had a previous diagnosis of GDM (7.5%) compared with women without an early OGTT (2.1%, p<0.01). Among women recorded as having pre-existing T2D, 89% (n=68) had an early HbA1c performed with mean HbA1c 7.6% (range 4.9%–14%) (table 1).

**Table 1:** Maternal characteristics according to women who had an early OGTT, women who did not and women with pre-existing T2D

	No early OGTT N=889	Early OGTT N=226	Pre-existing T2D N=76	P value
Maternal age at birth, years	24 (5.4)	26 (5.6)	30 (5.0)	<0.01
Nulliparous, n (%)	345 (39)	71 (31)	14 (18)	<0.01
1st BMI in pregnancy, kg/m^2^	25 (6.5)	27 (6.3)	31 (5.6)	0.001
Gestational weight gain, kg	7.7 (5.7)	8.1 (6.0)	5.5 (5.5)	0.295
Smoking in pregnancy, n (%)	476 (54)	104 (46)	33 (43)	0.045
Alcohol in pregnancy, n (%)	105 (12)	26 (12)	8 (11)	0.942
GDM in previous pregnancy, n (%)	19 (2.1)	17 (7.5)	9 (11)	<0.01
Gestational age at first recorded antenatal visit, weeks	10 (7, 17)	7 (6, 11)	9 (6, 15)	<0.01
Pre-existing hypertension prior to pregnancy, n (%)	12 (1.4)	4 (1.8)	5 (6.6)	0.004
Pre-existing renal disease prior to pregnancy, n (%)	24 (2.7)	8 (3.5)	9 (12)	<0.01
Pre-existing cardiac disease prior to pregnancy, n (%)	57 (6.4)	15 (6.6)	14 (18)	<0.01
Any pre-existing medical comorbidity, n (%)^a*^	85 (9.6)	26 (12)	24 (32)	<0.01

Women who had an early OGTT: BMI n=214, gestational weight gain n=220. Women who did not have an early OGTT: BMI n=810, gestational weight gain n=813. Women with pre-existing T2D: BMI n=74, gestational weight gain n=73.

* Any of pre-existing hypertension, renal disease or cardiac disease.

BMI, body mass index; early OGTT, an OGTT performed prior to 20 weeks gestation; GDM, gestational diabetes mellitus; OGTT, 75 g oral glucose tolerance test; T2D, type 2 diabetes.

#### Adverse pregnancy outcomes according to early screening status and pre-existing T2D

Compared with women who underwent guideline-directed early pregnancy OGTT, the combined adverse pregnancy outcome was more common among women with pre-existing T2D (89% vs 54%, adjusted OR 6.06 (95% CI 2.75 to 13.35)) and similar among women who did not undergo early OGTT (50%, adjusted OR 0.97 (95% CI 0.71 to 1.32)). Except for SGA, which occurred less frequently, each adverse pregnancy outcome was the most common among women with pre-existing T2D. Women with pre-existing T2D had a pre-term birth rate of 44% (n=33) and stillbirth rate of 9.3% (n=7) (table 2). Differences in the risk of each adverse pregnancy outcome between women with pre-existing T2D and other groups persisted after adjustment for maternal age, BMI, smoking status and parity, with the exception of SGA for which there was no difference in risk after adjustment. The crude rate of LGA was higher among women who had an early OGTT than those who did not, but the risk difference did not persist after adjustment (10.2% vs 5.8%, adjusted OR 0.73 (0.42 to 1.28)). There were no other differences in adverse pregnancy outcome rates between women who had an early OGTT and those who did not.

**Table 2:** Absolute pregnancy outcome rates and OR (95% CI, p value) for developing adverse pregnancy outcomes according to whether women had an early OGTT, did not have an early OGTT or had pre-existing T2D

	Absolute pregnancy outcomes			Unadjusted		Adjusted*	
	No early OGTT N=883	Early OGTT N=226	Pre-existing T2D N=75	No early OGTT N=883	Pre-existing T2D N=75	No early OGTT N=883	Pre-existing T2D N=75
Combined outcome†	437 (50%)	122 (54%)	67 (89%)	0.84 (0.62 to 1.11)	7.14 (3.28 to 15.55)‡	0.97 (0.71 to 1.32)	6.06 (2.75 to 13.35)‡
Large for gestational age	51 (5.8%)	23 (10.2%)	23 (31%)	0.54 (0.32 to 0.91)§	3.90 (2.03 to 7.50)‡	0.73 (0.42 to 1.28)	3.15 (1.54 to 6.43)‡
Small for gestational age	154 (18%)	31 (14%)	4 (5.3%)	1.33 (0.88 to 2.02)	0.35 (0.12 to 1.04)	1.20 (0.78 to 1.87)	0.43 (0.15 to 1.30)
Caesarean section	257 (29%)	65 (29%)	47 (63%)	1.02 (0.74 to 1.40)	4.16 (2.40 to 7.2)‡	1.22 (0.86 to 1.72)	3.50 (1.96 to 6.24)‡
Pre-eclampsia	39 (4.4%)	11 (4.9%)	9 (12%)	0.90 (0.46 to 1.79)	2.67 (1.05 to 6.70)§	0.94 (0.46 to 1.90)	2.84 (1.08 to 7.45)§
Pre-term delivery	123 (15%)	40 (18%)	33 (44%)	0.75 (0.51 to 1.11)	3.65 (2.07 to 6.46)‡	0.76 (0.50 to 1.16)	4.05 (2.20 to 7.47)‡
Special care nursery or NICU	193 (22%)	45 (20%)	46 (61%)	1.13 (0.78 to 1.62)	6.38 (3.61 to 11.26)‡	1.14 (0.78 to 1.69)	7.03 (3.86 to 12.79)‡
Stillbirth	11 (1.3%)	2 (0.9%)	7 (9.3%)	1.41 (0.31 to 6.41)	11.53 (2.34 to 56.80)‡	2.02 (0.25 to 16.11)	33.12 (3.58 to 40.60)‡

* Variables included in the adjusted model included maternal age, BMI, smoking during pregnancy and parity.

† Combined outcome is defined as having at least one of LGA, caesarean section, pre-eclampsia, pre-term delivery or admission to special care nursery or NICU.

‡ p<0.01.

§ p<0.05.

BMI, body mass index; early OGTT, an OGTT performed prior to 20 weeks gestation; GDM, gestational diabetes mellitus; LGA, large for gestational age; NICU, neonatal intensive care unit; OGTT, 75 g oral glucose tolerance test; T2D, type 2 diabetes.

Details of a subgroup analysis restricted to women diagnosed with GDM at some stage in pregnancy (n=154) is described in online supplemental table 1. In this subgroup analysis, women who were screened with an early OGTT, compared with women did not have an early OGTT, had a higher BMI (27 kg/m^2^ vs 24 kg/m^2^, p<0.01) and first presented for antenatal care earlier in gestation (8 weeks vs 11 weeks, p<0.01) but were of similar age (27 years vs 26 years, p=0.09). Among women with GDM, those who underwent an early OGTT were less likely to be managed with diet alone (42% vs 66%, p<0.01) and more likely to be managed with metformin alone (37% vs 19%, p=0.01), compared with women who did not undergo an early OGTT. There were no differences in terms of insulin management (online supplemental table 1). There were no differences in pregnancy outcomes between those who underwent early OGTT screening and those who did not (online supplemental table 2).

## Discussion

This retrospective study of pregnancies among Aboriginal women in the remote NT aimed to determine uptake of hyperglycaemia in pregnancy screening and pregnancy outcomes in women who did and did not have an early OGTT and those with pre-existing T2D. We report four key findings. First, while there were low rates of screening as per recommendations, most women had at least some form of glycaemic screening during pregnancy. Second, women who had early OGTT screening had a higher BMI, were older, had higher rates of previous GDM diagnosis, and presented for antenatal care earlier in gestation. Third, women with an early OGTT had higher crude rates of LGA compared with women without an early OGTT, that did not remain significant after adjustment for covariates and was likely driven by the higher BMI in the group of women who had an early OGTT. Contrary to our initial hypothesis, both groups had similar rates of other pregnancy outcomes. Fourth, women with pre-existing T2D had markedly higher rates of adverse pregnancy outcomes compared with women without pre-existing T2D.

Rates of guideline-directed GDM screening were low in our study population, with only 14% of pregnancies receiving the full recommended screening schedule. Half of the pregnancies in our cohort had routine screening with an OGTT only, and approximately a quarter had an HbA1c only. Despite current local guidelines recommending universal screening with an early pregnancy OGTT for Aboriginal women,^17^ this was only undertaken in 20% of pregnancies. Reassuringly, only a small percentage of pregnancies had no form of glycaemic testing. Previous small Australian studies investigating GDM screening had similar findings, including only 17% of Aboriginal women birthing at Alice Springs Hospital having had an early OGTT (^20 21^) and 51% of women in remote Western Australia having any OGTT screening.^22^ While our study did not explore potential reasons for low OGTT uptake, previous studies have reported poor tolerance of the glucose drink (the need to fast, the unpleasantness of the test and time-consuming nature) particularly in the first trimester of pregnancy, and lack of both transport and childcare as being barriers to completion.^23 24^ In addition, we hypothesise that health professionals are either not prioritising an early OGTT, in order to focus on a routine OGTT, or not prioritising an OGTT at all, instead relying on an HbA1c given the difficulties in performing an OGTT. There are additional challenges for women living remotely, including accessible and appropriately coordinated healthcare, the high turnover and shortage of health professionals, and socioeconomic disadvantage.^25^ In our setting, it is important to note that the reported GDM prevalence of 14% is likely an underestimate due to delayed OGTT sample processing in remote settings, which can lead to glucose degradation from glycolysis.^26^

Our study identified that women screened with an early OGTT had a higher BMI, were older, had higher rates of previous GDM diagnoses, and presented for antenatal care earlier in gestation than those who did not have an early OGTT. This is consistent with the literature.^27^,^29^ These findings could indicate that health professionals are selecting patients with additional risk factors for early screening rather than using ethnicity alone as an indication. Additionally, earlier gestational age at first antenatal visit likely indicates increased engagement with the healthcare system and greater opportunities for healthcare professionals to discuss and complete GDM screening.

Few trials have investigated the efficacy of early pregnancy screening and treatment for GDM in women with risk factors. Some observational studies suggest that early GDM has worse outcomes than GDM diagnosed later in pregnancy.^29^ In retrospective cohorts where there was no difference, or even improvement, in rates of adverse pregnancy outcomes observed with early GDM, it is not possible to delineate whether there was no difference in baseline risk or whether risk was ameliorated by early treatment.^28^,^30^ The Early Gestational Diabetes Screening in the Gravid Obese Woman randomised trial in the USA found no improvement in perinatal outcomes with early screening.^31^ However, this study was limited by having a relatively small number of women with GDM and there was only a 3-week difference in the average gestational age at GDM diagnosis between the groups. A large preintervention and postintervention study in the USA suggested that women with obesity may benefit from early screening.^30^ The recent Treatment Of BoOking Gestational diabetes Mellitus randomised controlled trial evaluated pregnancy outcomes following treatment of early GDM.^32^ The study reported that treatment of early GDM led to a modestly lower incidence of a composite of adverse neonatal outcomes than no early treatment. Generalisability of international studies and studies in non-Aboriginal women to our cohort may be limited due to heterogeneity in patient groups, screening methods, diagnostic criteria and treatment regimens.

We hypothesised that women who were not screened for GDM in early pregnancy might be at particularly high risk for adverse pregnancy outcomes, suggesting the need to focus our attention on improving screening uptake. However, we reported similar rates of overall adverse pregnancy outcomes among women who were and were not screened in early pregnancy. LGA rates were, in fact, higher among women who had an early pregnancy OGTT that could be attributed to women with early screening having a higher BMI and the known relationship between higher rates of LGA and higher maternal BMI.^31^ This is supported by the finding that when adjusted for BMI, the odds of developing LGA did not persist. Perhaps the lack of difference in adverse pregnancy outcomes was because the women prioritised for early screening by health professionals had a higher baseline risk. Furthermore, despite 16.4% of women undergoing early OGTT having hyperglycaemia in pregnancy, this represented a small number of women (n=37) in the context of low early pregnancy OGTT screening rates. So treatment differed for only a small number of women. The high rate of pre-existing T2D, with few cases of overt DIP detected, suggests good diabetes screening uptake outside of pregnancy.

Since colonisation, Aboriginal and Torres Strait Islander peoples continue to experience social and economic inequities, including suboptimal housing, psycho-social stressors and a lack of economic and occupational opportunities.^33^ Consequently, there is a high burden of youth-onset T2D for Aboriginal peoples as reflected in our observed very high prevalence of women diagnosed with T2D prior to pregnancy.^34 35^ In addition, our observed high rates of adverse pregnancy outcomes for women with pre-existing T2D are consistent with the literature^36^ and highlight significant disparities for these women. Of particular concern is the high pre-term birth rate of 44% and high stillbirth rate of 9% which is markedly higher than the general Australian stillbirth rate of 0.77%.^37^

Limitations of our study include that it is a retrospective study and there may be missing data on screening, although manual chart review of all cases without OGTT screening was reviewed to minimise this risk. Analyses did not account for the fact that women diagnosed with GDM had access to standard treatment, or that there was likely variability in concordance with treatment. Strengths include the uniqueness of this cohort for its high number of women with pre-existing T2D, meaning we were able to highlight the concerningly high rates of adverse pregnancy outcomes. Additional strengths of this study include exploring health outcomes specifically in Aboriginal women and a large study cohort which included BMI data. As our study occurred after the adoption of the International Association of Diabetes and Pregnancy Study Groups guidelines,^38^ and prior to the COVID-19 pandemic, all women in this study were screened and diagnosed with GDM according to the same guidelines.

## Conclusion

Despite local recommendations for early pregnancy OGTT screening, uptake among Aboriginal women in remote NT communities is low. Nevertheless, there was no meaningful increase in risk of adverse pregnancy outcomes in women who did not undergo early pregnancy screening with an OGTT compared with those who did. Rates of pregnancy HbA1c screening were higher, likely reflecting greater acceptability and convenience of this test. Further research is needed to clarify the role of HbA1c testing in early pregnancy, including appropriate diagnostic thresholds. Data on the sensitivity and specificity of HbA1c in this context remain limited. The validity of HbA1c as a screening approach, and its relationship to gestational diabetes screening and pregnancy outcomes, will be examined in a subsequent analysis.

The incidence of adverse pregnancy outcomes, including stillbirth and pre-term birth, reported in women with T2D is very concerning. There is an urgent need to strengthen care for these women. Research examining potential barriers and strategies on how best to support T2D care for Aboriginal mothers and families in the remote NT could be instrumental in improving pregnancy outcomes for current and future generations.

## Supplementary material

10.1136/bmjopen-2025-110242 online supplemental file 1

## Data Availability

Data are available upon reasonable request.
