# Supplementary material for: Screening for hyperglycaemia in pregnancy and pregnancy outcomes among Aboriginal women in remote communities of the Northern Territory, Australia: a retrospective cohort study
Source: BMJ Open. 2026 May 19;16(5):e110242. doi: 10.1136/bmjopen-2025-110242 (PMC13202063; doi:10.1136/bmjopen-2025-110242)
Supplement: online supplemental file 1 [file bmjopen-16-5-s001.docx]

**Supplementary Table 1: Maternal characteristics among women** **diagnosed with GDM stratified by whether or not they had an early OGTT**

|  | **No early OGTT**  **N=95** | **Early OGTT**  **N=59 ^a^** | **P-value** |
| --- | --- | --- | --- |
| Maternal age at birth, years | 26 (5.4) | 27 (5.6) | 0.09 |
| Nulliparous, n (%) | 33 (35) | 16 (27) | 0.32 |
| 1^st^ BMI in pregnancy, kg/m^2^ | 24 (5.3) | 27 (5.9) | **<0.01** |
| Gestational weight gain, kg | 8.4 (4.8) | 6.7 (5.2) | 0.05 |
| Smoking in pregnancy, n (%) | 54 (57) | 27 (46) | 0.18 |
| Alcohol in pregnancy, n (%) | 9 (10) | 5 (9) | 0.83 |
| GDM in previous pregnancy, n (%) | 5 (5.3) | 12 (20) | **<0.01** |
| Gestational age at first recorded antenatal visit, weeks | 11 [7, 19] | 8 [6, 10] | **<0.01** |
| Pre-existing hypertension prior to pregnancy, n (%) | 1 (1.1) | 1 (1.7) | 0.73 |
| Pre-existing renal disease prior to pregnancy, n (%) | 5 (5.3) | 4 (6.8) | 0.70 |
| Pre-existing cardiac disease prior to pregnancy n (%) | 10 (11) | 6 (10) | 0.94 |
| Any pre-existing medical comorbidity, n (%)^b^ | 14 (15) | 11 (19) | 0.52 |
| **Diabetes management in pregnancy** |  |  |  |
| Diet only | 63 (66) | 25 (42) | **<0.01** |
| Metformin only | 18 (19) | 22 (37) | **0.01** |
| Insulin and metformin | 12 (13) | 11 (19) | 0.31 |
| Insulin only | 2 (2) | 1 (1.7) | 0.86 |

a. Including 37 women who were diagnosed with GDM on an early OGTT and 22 women who had negative early OGTT and diagnosed with GDM on a routine OGTT

b. Any of pre-existing hypertension, renal disease or cardiac disease.

Women who had an early OGTT: BMI n=92, Gestational weight gain n=90. Women who did not have an early OGTT: BMI n=58, Gestational weight gain n=56. Women with pre-existing T2D: BMI n=74, Gestational weight gain n=73.

OGTT: 75g oral glucose tolerance test, GDM: gestational diabetes, Early OGTT: an OGTT performed prior to 20 weeks gestation**Supplementary Table 2: Absolute pregnancy outcome rates and odds ratio (95% CI, p-value) for developing adverse pregnancy outcomes among women diagnosed with GDM stratified by whether or not they had an early OGTT**

|  | **Absolute pregnancy outcomes** | | **Unadjusted** | | **Adjusted^a^** | |
| --- | --- | --- | --- | --- | --- | --- |
|  | **No early OGTT**  **N=95** | **Early OGTT**  **N=59** | **No early OGTT**  **N=95** | **P value** | **No early OGTT**  **N=95** | **P value** |
| Combined outcome^b^ | 59 (62%) | 36 (61%) | 0.88 (0.46-1.70) | 0.71 | 0.85 (0.38-3.00) | 0.64 |
| Large for gestational age | 10 (11%) | 9 (15%) | 0.68 (0.26-1.79) | 0.43 | 1.06 (0.83-5.48) | 0.91 |
| Small for gestational age | 10 (11%) | 7 (12%) | 0.88 (0.31-2.44) | 0.80 | 0.71 (0.24-2.12) | 0.54 |
| Caesarean section | 43(45%) | 21 (36%) | 1.50 (0.77-2.92) | 0.24 | 1.56 (0.77-3.18) | 0.21 |
| Preeclampsia | 3 (3.2%) | 3 (5.1%) | 0.61 (0.12-3.12) | 0.55 | 0.56 (0.11-3.00) | 0.50 |
| Pre-term delivery | 14 (15%) | 10 (17%) | 0.85 (0.35-2.05) | 0.71 | 0.56 (0.22-1.45) | 0.23 |
| Special care nursery or NICU | 27 (28%) | 14 (24%) | 1.28 (0.60-2.69) | 0.52 | 1.07 (0.48-2.38) | 0.88 |
| Stillbirth | 0 | 0 |  |  |  |  |

1. Variables included in the adjusted model included maternal age, BMI, smoking during pregnancy and parity.
2. Combined outcome is defined as having at least one of LGA, caesarean section, preeclampsia, pre-term delivery or admission to special care nursery or NICU.

OGTT: 75g oral glucose tolerance test, GDM: gestational diabetes, Early OGTT: an OGTT performed prior to 20 weeks gestation, NICU: neonatal intensive care unit.
