# Supplementary material for: Screening for hyperglycaemia in pregnancy and pregnancy outcomes among Aboriginal women in remote communities of the Northern Territory, Australia: a retrospective cohort study
Source: BMJ Open. 2026 May 19;16(5):e110242. doi: 10.1136/bmjopen-2025-110242 (PMC13202063; doi:10.1136/bmjopen-2025-110242)
Supplement: Supplementary file 2 [file bmjopen-2025-110242.reviewer_comments.pdf]

PEER REVIEW HISTORY

BMJ Open publishes all reviews undertaken for accepted manuscripts. Reviewers are asked to complete a checklist review form and are provided with free text boxes to elaborate on their assessment. These free text comments are reproduced below.

ARTICLE DETAILS

Title (Provisional)

A retrospective cohort study of screening for hyperglycaemia in pregnancy and pregnancy outcomes among Aboriginal women in remote communities of the Northern Territory, Australia

Authors

Wood, Anna; O'hara, Chloe; Joyce-Tubb, Ariella; Webster, Vanya; Wicks, Mary; Van Dokkum, Paula; Maple-Brown, Louise; Hare, Matthew J. L.

VERSION 1 - REVIEW

**Reviewer**	**1**
**Name**	**Nolan, Christopher**
**Affiliation**	**Australian National University, Medical School**
**Date**	**14-Oct-2025**
**COI**	**None**

This is a highly worthwhile retrospective analysis of in pregnancy hyperglycaemia screening practices and pregnancy outcomes in remote Indigenous Australian communities of the Northern Territory of Australia.

Most importantly it shows very high rates of serious adverse outcomes in pregnancies of the women with pre-existing type 2 diabetes.

I have some questions that the authors should consider to improve the quality of the manuscript.

1. The overall rates of GDM in the community is reported to be 13% which seems low considering the 6.4% rate of pre-existing diabetes. Could this be falsely low as a result of the OGTT methodology? It has been shown that OGTT samples collected in NaF tubes that are a distance from the laboratory can give falsely low glucose levels substantially reducing GDM diagnosis rates. What was the pre-analytical processes used for the OGTTs and could this have contributed to some of the findings?

2. Early pregnancy HbA1c was tested in 69% of all pregnancies without pre-existing diabetes, but there has been little analysis of the findings reported. Of those women with complete

and incomplete screening, how many had early HbA1c measurements? Could an early HbA1c have been used as an alternative to early OGTT in some of the remote communities? Could further data on early HbA1c be added to the manuscript and could this be discussed?

**Reviewer**	**2**
**Name**	**Soepnel, Larske M.**
**Affiliation**	**University of the Witwatersrand Johannesburg**
**Date**	**11-Nov-2025**
**COI**	**None**

Thank you for the opportunity to review this manuscript, which investigated the rates of screening for hyperglycaemia in pregnancy among Aboriginal women in remote communities in the Northern Territory, Australia. The manuscript provides important insights into health systems use in this understudied context. I recommend some revisions prior to publication.

1. My main concern is that the chosen group comparisons is not well justified. In particular, it is not clear why authors chose to compare outcomes of screening groups regardless of their 'hyperglycaemia in pregnancy' status, since the majority of each group does not have hyperglycaemia, and therefore would not have any conceptual basis to expect different pregnancy outcomes based on screening method. (in other words, if you don't have hyperglycaemia, it may make no difference whether you're screened early or later or not at all, but it may make a big difference for those with the condition). This likely explains why there were no differences in pregnancy outcomes between the groups. To me, a more logical comparison would be to compare pregnancy outcomes between early/no early screening among participants with hyperglycaemia.

For example, in the conclusion: "Nevertheless, there was no meaningful increase in risk of adverse pregnancy outcomes in women who did not undergo early pregnancy screening with an OGTT compared to those that did." The implications/meaning of this finding among a group of majority non-GDM participants is unclear to me. Since this is central to the manuscript aim, it either requires some amendment or clear justification/rationale.

Moreover, what was the reasoning behind grouping 'standard OGTT' together with 'hba1c testing' under 'incomplete'? To my understanding, the standard OGTT is still evidence-based and an important recommendation, whereas using HbA1c as a diagnostic tool (in pregnancy and without) is more contested due to limited sensitivity and specificity. This means that the 'incomplete' group is very wide and encompasses conceptually distinct methods, limiting the meaning derived from comparisons made to this group.

In addition, there seemed to be some inconsistency in the terminology used for the groups. While the methods and descriptive part of the results seemed to compare complete vs

incomplete vs no screening, the rest of the analyses and discussion refer to early screening vs no early screening. I would suggest that the authors choose a single grouping, to improve the clarity of the manuscript aim and message. Alternatively, please clarify and justify the different terminology used.

I also question the motivation behind comparing women with pre-existing T2D to those with different screening methods. It has been established that women with T2D often have worse outcomes (as the authors also mention in the discussion), let alone when comparing to the two groups that consist of a majority of participants with no hyperglycaemia first discovered in pregnancy at all. What was the rationale behind this comparison? What does it tell us about the screening in this group?

On a related, but minor note, in the abstract, could the authors adapt the phasing to clarify which groups are compared to which other groups? This is currently unclear from the abstract.

2. Did early screening also lead to early/comprehensive management or treatment? If not, no improvement in outcomes should be expected. Did the authors have access to treatment data? If not, I think this is an important limitation to address in more depth. If the authors do have access to this data, I would recommend that they more clearly model (even as a figure) a screening/treatment cascade for the included participants, which could help identify where in the process the main pitfalls to successful identification and management lie. This may provide more insight that the current group comparisons.

3. Timing of the first antenatal visit likely has an independent impact on pregnancy outcome. How did authors treat this in the models? I suggest exploring how this variable impacts adverse pregnancy outcomes as well as screening outcomes.

4. Please clarify how repeat pregnancies were handled – am I correct that there were 50 cases? Since these are not independent observations, how did the authors treat these in the statistical analysis?

5. When reporting the prevalence results, it seems like the denominator for Hyperglycaemia in pregnancy was all eligible pregnancies (eg. 1078) but for reporting DIP/GDM it was those who underwent an OGTT (eg. 682); please clarify and amend this, reporting prevalence with consistent denominators?

6. Please clarify what changes have been made to the screening recommendations since the study period? What impact does this have on the implications of your results going forward?

7. In the discussion, the third main finding "Third, women with an early OGTT…": this did not remain significant after adjustment for covariates and is very likely driven by the higher BMI in the early OGTT group. I suggest amending the phrasing of this finding accordingly.

8. I suggest adding a few sentences to the discussion on the sensitivity/specificity of HbA1c for hyperglycaemia in pregnancy. Is there any data on this among Aboriginal women? This is

important to consider prior to recommending that HbA1c should be further tested/considered for diagnosis.

9. In the conclusion: "… is arguably a greater priority than GDM screening uptake": I don't think you can conclude this based on the presented results, since we do not know about treatment, differences in pregnancy outcomes among those with hyperglycaemia, etc.

Minor edits:

1. In the introduction, page 3-4, "Uptake of early screening with an OGTT is suspected to be low" – what is this 'suspicion' based on?

2. I would recommend authors use the REporting of studies Conducted using Observational routinely collected Data (RECORD) extension of the STROBE checklist.

3. In the results, authors report "similarly high rates of smoking" (page 8), but the p-value indicates significant difference between groups. Please rephrase to clarify.

VERSION 1 - AUTHOR RESPONSE

Reviewer 1

1. **The overall rates of GDM in the community is reported to be 13% which seems low considering the 6.4% rate of pre-existing diabetes. Could this be falsely low as a result of the OGTT methodology? It has been shown that OGTT samples collected in NaF tubes that are a distance from the laboratory can give falsely low glucose levels substantially reducing GDM diagnosis rates. What was the pre-analytical processes used for the OGTTs and could this have contributed to some of the findings?**

Thank you for highlighting this important point. We do agree that we are very likely under-diagnosing GDM due to OGTT methodology. In our context there are large distances and delays to laboratory analysis. This was highlighted in the ORCHID study[1] conducted in regional and remote Western Australia that explored the potential impact of glycolysis on GDM diagnosis. They reported that most routine OGTT samples were analysed more than four hours post-fasting collection (median 5.0h, range 2.3 to 124h) potentially reducing glucose levels due to glycolysis. The investigators evaluated alternative sample handling protocols in a sample of participants and reported a 62% under-diagnosis of GDM with the standard protocol (routine room temperature (RT) fluoride-oxalate samples ($FLOX^{RT}$)). Research into the impacts of pre-analytical process on rates of GDM in the Northern Territory has not yet been conducted and is a priority that needs to be addressed. Given routine use of NaF tubes and long distances to laboratories, it is likely that rates of under-diagnosis are similar to those observed in remote Western Australia.

The incidence of GDM was not the focus of this paper and our comparative analyses of pregnancy outcomes focus on screening (rather than diagnosis) status. Therefore, we feel that the main results of our study are unlikely to have been meaningfully affected by this issue. Nevertheless, we agree that the issue is very relevant to the subject of this manuscript and have therefore added the following to the discussion:

"In our setting, it is important to note that the reported GDM prevalence of 14% is likely an underestimate due to delayed OGTT sample processing in remote settings, which can lead to glucose degradation from glycolysis (25)".

1. Jamieson EL et al, Int J Environ Res and Public Health, 2019

2. Early pregnancy HbA1c was tested in 69% of all pregnancies without pre-existing diabetes, but there has been little analysis of the findings reported. Of those women with complete and incomplete screening, how many had early HbA1c measurements? Could an early HbA1c have been used as an alternative to early OGTT in some of the remote communities? Could further data on early HbA1c be added to the manuscript and could this be discussed?

Thank you, we agree that these are important questions. Whether an HbA1c can be considered as an alternative screen for hyperglycaemia in pregnancy, especially in the remote context, is an important consideration. Among the 774 women (69%) with HbA1c < 20 weeks, 182/774 (24%) also had OGTT <20 weeks and 475/774 (62%) also had OGTT ≥20 weeks. The ability of HbA1c to diagnosis GDM and how it fares in its ability to predict adverse pregnancy outcomes is the focus of a second paper, which is under preparation. We did initially consider putting it all in one manuscript but want to look at associations between HbA1c (at various thresholds) with clinical pregnancy outcomes and subsequent OGTT results. In the end, the authors decided that there was too much information and separate background evidence to discuss for one paper.

Reviewer: 2

1. My main concern is that the chosen group comparisons is not well justified. In particular, it is not clear why authors chose to compare outcomes of screening groups regardless of their 'hyperglycaemia in pregnancy' status, since the majority of each group does not have hyperglycaemia, and therefore would not have any conceptual basis to expect different pregnancy outcomes based on screening method. (in other words, if you don't have hyperglycaemia, it may make no difference whether you're screened early or later or not at all, but it may make a big difference for those with the condition). This likely explains why there were no differences in pregnancy outcomes between the groups. To me, a more logical comparison would be to compare pregnancy outcomes between early/no early screening among participants with hyperglycaemia.

For example, in the conclusion: "Nevertheless, there was no meaningful increase in risk of adverse pregnancy outcomes in women who did not undergo early pregnancy screening with an OGTT compared to those that did." The implications/meaning of this finding among a group of majority non-GDM participants is unclear to me. Since this is central to the manuscript aim, it either requires some amendment or clear justification/rationale.

Thank you, you raise an important point. The reason we focused on screening is that the paper was framed to focus on the hypothesis that women who didn't undergo screening were likely to be a high risk group and hence we wanted to look at outcomes in that group to strengthen advocacy for screening strategies. We anticipated that missing screening would be marker of risk because this cohort is likely less engaged with health care and face barriers to access. We did not expect to indeed find no differences in outcomes between those who were and were not screened.

However, we do acknowledge your concerns and to address these concerns, we have conducted a sub-group analysis among women who received a diagnosis of GDM - see Supplementary table 1 and 2 and added text added to the Results section, page 8.

"Details of a subgroup analysis restricted to women diagnosed with GDM at some stage in pregnancy (n=154) is described in Supplementary Table 1. …

There were no differences in pregnancy outcomes between those who underwent early OGTT screening and those who did not (Supplementary table 2)."

Moreover, what was the reasoning behind grouping 'standard OGTT' together with 'hba1c testing' under 'incomplete'? To my understanding, the standard OGTT is still evidence-based and an important recommendation, whereas using HbA1c as a diagnostic tool (in pregnancy and without) is more contested due to limited sensitivity and specificity. This means that the 'incomplete' group is very wide and encompasses conceptually distinct methods, limiting the meaning derived from comparisons made to this group.

In addition, there seemed to be some inconsistency in the terminology used for the groups. While the methods and descriptive part of the results seemed to compare complete vs incomplete vs no screening, the rest of the analyses and discussion refer to early screening vs no early screening. I would suggest that the authors choose a single grouping, to improve the clarity of the manuscript aim and message. Alternatively, please clarify and justify the different terminology used.

Thank you for highlighting this - we do agree that the terminology used was confusing and recognise that Table 1 traditionally describes the cohort, whereby we have presented Table 1 as the different screening types and Table 2 as the cohort. In response to your comments, we have now removed Table 1 and the confusing terminology around complete and incomplete screening.

Table 1 has been replaced with a figure (Figure 2) that we think better captures the various screening that is occurring. We wanted to provide a comprehensive view of what testing is being done in our setting as this is important for local services and policy development.

Please review updated abstract, methods, results and figure 2:

Figure 2: **Rates of hyperglycaemia screening for Aboriginal women in remote communities in the NT between 2017-19 (n=1115)**

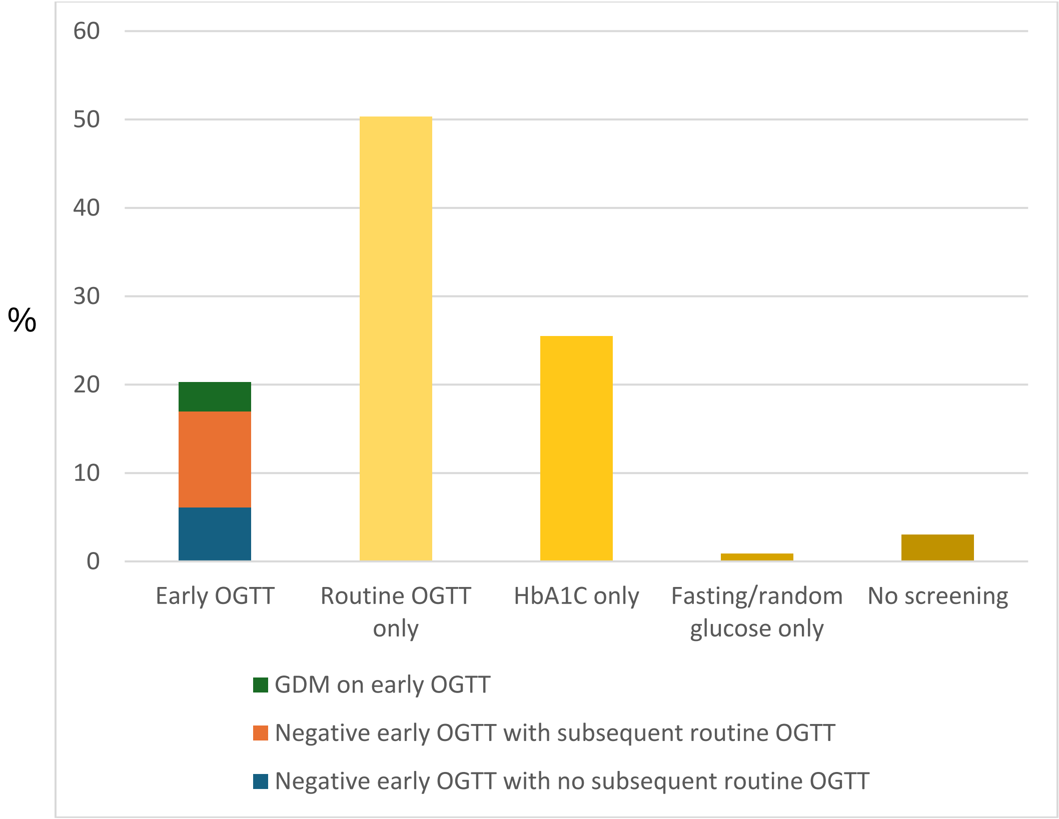

I also question the motivation behind comparing women with pre-existing T2D to those with different screening methods. It has been established that women with T2D often have worse outcomes (as the authors also mention in the discussion), let alone when comparing to the two groups that consist of a majority of participants with no hyperglycaemia first discovered in pregnancy at all. What was the rationale behind this comparison? What does it tell us about the screening in this group?

Thank you for highlighting this and we completely understand your comments. The comparison against T2D is obviously not novel scientific information. The rationale for presenting outcomes for women with T2D was to provide context to help with determination of local priorities for policy and practice changes. Given our high rates of T2D in pregnancy, if we were to omit this group, we would be missing an important perspective. There is much discussion regarding how to best screen for and diagnose GDM. Meanwhile, we have very high rates of pre-existing T2D in pregnancy. This study shows that despite universal free access to health care in Australia, we are still seeing terrible clinical outcomes in this context. We felt it was important to highlight these inequitable outcomes to help inform discussions regarding priorities for screening and management of hyperglycaemia in pregnancy. We have included a sub-analysis, as described above, comparing outcomes among women with GDM according to whether or not they underwent early OGTT screening.

On a related, but minor note, in the abstract, could the authors adapt the phasing to clarify which groups are compared to which other groups? This is currently unclear from the abstract.

This has been addressed with changes in the abstract as per comment above, thanks.

2. Did early screening also lead to early/comprehensive management or treatment? If not, no improvement in outcomes should be expected. Did the authors have access to treatment data? If not, I think this is an important limitation to address in more depth. If the authors do have access to this data, I would recommend that they more clearly model (even as a figure) a

screening/treatment cascade for the included participants, which could help identify where in the process the main pitfalls to successful identification and management lie. This may provide more insight that the current group comparisons.

Our hypothesis and aim were not to show that early screening is beneficial. This is because we agree with you that there is an inherent limitation of a retrospective design. The potential benefits of early GDM screening are better addressed in a randomised control trial. Instead, we framed our aim and hypothesis around describing a real life cohort being recommended screening to help our local priorities.

In terms of medication use, we do have data on treatment, however, as this is a real-word retrospective review, we do not have details on treatment cascades or on adherence to treatments. We have now added treatment information to Supplementary Table 1 and included the following in the text, page 8 regarding treatment:

"Among women with GDM, those who underwent an early OGTT were less likely to be managed with diet alone (42% vs 66%, $p<0.01$) and more likely to me managed with metformin alone (37% vs 19%, $p = 0.01$), compared to women who did not undergo an early OGTT. There were no differences in terms of insulin management (Supplementary Table 1)."

3. Timing of the first antenatal visit likely has an independent impact on pregnancy outcome. How did authors treat this in the models? I suggest exploring how this variable impacts adverse pregnancy outcomes as well as screening outcomes.

We found that women who had an early OGTT had an earlier first visit and we think these women are likely more engaged and therefore have more opportunities for screening. We didn't include this variable in the model. Variables were selected a priori and due to limited events and risk of overfitting models, a parsimonious model without too many variables was chosen. When we conducted a sensitivity analysis including this variable in the model, it does not meaningfully change results.

4. Please clarify how repeat pregnancies were handled – am I correct that there were 50 cases? Since these are not independent observations, how did the authors treat these in the statistical analysis?

Yes you are correct. As described in the methods section, cluster-robust standard errors were used to account for lack of independence in the sample due to a small number of women who had multiple pregnancies within the study period.

5. When reporting the prevalence results, it seems like the denominator for Hyperglycaemia in pregnancy was all eligible pregnancies (eg. 1078) but for reporting DIP/GDM it was those who underwent an OGTT (eg. 682); please clarify and amend this, reporting prevalence with consistent denominators?

Thank you for bringing up this point, and we acknowledge that the different denominators could be clearer.

- The denominator for hyperglycaemia is 1115 (all pregnancies excluding T2D)
- The denominator for routine OGTT is 1078 (excluding women diagnosed with GDM on an early OGTT: 1115 minus 37)

In our results section, we describe the proportion of those who have an OGTT who had GDM, hence the denominator of 226 for early OGTT and 682 for routine OGTT. We have now changed the results and terminology to have a consistent denominator for each section:

Page 7:

"Hyperglycaemia in pregnancy was newly detected in 3.3% (n/N = 37/1115) of all pregnancies at early screening, with 2.8% (n/N = 31/1115) meeting criteria for GDM and 0.5% (n/N = 6/1115) meeting criteria for overt DIP."

A routine OGTT was undertaken in 63% (n/N = 682/1078) of eligible pregnancies. Hyperglycaemia in pregnancy was detected in 11% (n/N = 117/1078), with 9% (n/N = 98/1078) meeting criteria for GDM and 1.3% (n/N = 14/1078) meeting criteria for overt DIP."

6. Please clarify what changes have been made to the screening recommendations since the study period? What impact does this have on the implications of your results going forward?

Since the study period, the Australasian recommendations for screening for GDM have changed (Sweeting et al Med J Aust. 2025 Aug 4;223(3):161-167.doi: 10.5694/mja2.52696). Rather than an early OGTT being recommended for all Aboriginal and Torres Strait Islander women, an early OGTT is now only recommended if there is a history of GDM or an early pregnancy HbA1C 6.0-6.4%.

Our results have been presented according to the current guidelines at the time the study was conducted. The change in national guidance does not directly affect interpretation of our results. The Northern Territory has recently adopted the new national guidance for local policy. Our study could be interpreted as supporting this change in screening approach, with less emphasis on early OGTT but with a safety net of routine HbA1c in early pregnancy to screen for previously undiagnosed per-existing diabetes. Given our senior author's direct involvement in preparation and implementation of the national guidelines, we would prefer not to make inferences in our manuscript relating to the new guidelines which might be perceived as biased.

7. In the discussion, the third main finding "Third, women with an early OGTT…": this did not remain significant after adjustment for covariates and is very likely driven by the higher BMI in the early OGTT group. I suggest amending the phrasing of this finding accordingly.

Thank you, we have changed the sentence to emphasise this: Page 8, now reads as:

"Third, women with an early OGTT had higher crude rates of LGA compared to women without an early OGTT, that did not remain significant after adjustment for covariates and was likely driven by the higher BMI in the group of women who had an early OGTT."

8. I suggest adding a few sentences to the discussion on the sensitivity/specificity of HbA1c for hyperglycaemia in pregnancy. Is there any data on this among Aboriginal women? This is important to consider prior to recommending that HbA1c should be further tested/considered for diagnosis.

There are no readily available data on the sensitivity and specificity of HbA1c for the detection of hyperglycaemia in pregnancy. This is an important consideration and will be the focus of future

research with this dataset, including examination of associations between HbA1c (at various threshold) with pregnancy outcomes.

9. In the conclusion: "… is arguably a greater priority than GDM screening uptake": I don't think you can conclude this based on the presented results, since we do not know about treatment, differences in pregnancy outcomes among those with hyperglycaemia, etc.

Thank you for this comment. We have removed this phrase from the conclusion.

Minor edits:
1. In the introduction, page 3-4, "Uptake of early screening with an OGTT is suspected to be low" – what is this 'suspicion' based on?

Thank you for noting the lack of reference with this statement, this was an oversite. We have now included a reference that supports this statement and slightly expanded this sentence:

"Given the low uptake of early screening with an OGTT described in other remote settings (ref), uptake in our context is suspected to be low, and whether alternate tests such as an HbA1c or isolated plasma glucose levels (fasting or random) are being used instead is not known."

Ref: Jamieson EL, Spry EP, Kirke AB, Atkinson DN, Marley JV. **Real-world screening for diabetes in early pregnancy: Improved screening uptake using universal glycated haemoglobin.** Prim Care Diabetes. 2021 Dec;15(6):995–1001. doi:10.1016/j.pcd.2021.09.011

2. I would recommend authors use the REporting of studies Conducted using Observational routinely collected Data (RECORD) extension of the STROBE checklist.

Thankyou- in addition to the STROBE checklist initially submitted, please see the RECORD checklist attached.

3. In the results, authors report "similarly high rates of smoking" (page 8), but the p-value indicates significant difference between groups. Please rephrase to clarify.

Thank you for picking this up, we have removed the word 'similarly' as we agree that while there were high rates of smoking across the board, there were slightly higher rates (54%) for women with no early OGTT (46% for those with an OGTT and 43% for those with T2D).

VERSION 2 - REVIEW

**Reviewer**	**1**
**Name**	**Nolan, Christopher**
**Affiliation**	**Australian National University, Medical School**
**Date**	**09-Feb-2026**
**COI**	

Thank you for improving the manuscript.

Major comments:

The analyses performed are predominantly around 3 main groups of pregnant women- those with pre-existing T2D diabetes, those without pre-existing diabetes who were or were not screened for GDM according to guidelines (early + late OGTT (if early OGTT was negative)). Clarity of the manuscript could be improved if the focus remains on these 3 groups, as mention of screening with HbA1c and fasting or random glucose reduces clarity.

Also there is no mention as to whether HbA1c or fasting/random blood glucose measures were acted on in any way- were cut-offs used for action or diagnosis of GDM etc. When were these performed early or late? There was 1 person who had a HbA1c diagnostic of overt diabetes in pregnancy- presumably treated. Again because of lack of detailed analyses of these other tests of glycaemia- much less focus should be placed on these in the manuscript.

Suggest- remove mention of HbA1c, fasting/random glucose from abstract.

For Figure 2 - reformat without HbA1c and fasting/random blood glucose.

Page 7- 2nd and 3rd paragraphs. As well as reporting the % of the whole cohort having hyperglycemia in pregnancy (GDM and ODIP) - can the % of those tested with an OGTT early and late also be included.

The subgroup analysis is helpful. Mention in the results section that the group that underwent early OGTTs were older with higher BMIs needs to be included.

**Reviewer**	**2**
**Name**	**Soepnel, Larske M.**
**Affiliation**	**University of the Witwatersrand Johannesburg**
**Date**	**26-Jan-2026**
**COI**	

Thank you for the opportunity to review the revised manuscript. Well done to the authors on an important and well-written piece of research.

I had two minor suggestions for the authors:

1. In my version of the manuscript (with track changes) there was a reference missing in the Introduction, page 4 (after, "more broadly in the literature").

2. I would suggest, based on the authors' response to both reviewers comments regarding HbA1c as a screening method, adding these considerations in a bit more detail into the discussion. For example, a sentence explaining that there are not adequate sensitivity/specificity data currently available for the context, and that the research group is working on a follow-up manuscript to contribute to this lack of knowledge (just a bit more

specific than the current sentence in the conclusion, which currently reads: "Rates of pregnancy HbA1c screening were higher, likely reflecting greater acceptability and convenience of this test. Future research should further evaluate the role of HbA1c testing in early pregnancy in this context, including consideration of diagnostic thresholds.").

VERSION 2 - AUTHOR RESPONSE

Reviewer: 1

1. **The analyses performed are predominantly around 3 main groups of pregnant women- those with pre-existing T2D diabetes, those without pre-existing diabetes who were or were not screened for GDM according to guidelines (early + late OGTT (if early OGTT was negative)). Clarity of the manuscript could be improved if the focus remains on these 3 groups, as mention of screening with HbA1c and fasting or random glucose reduces clarity.**
   Also there is no mention as to whether HbA1c or fasting/random blood glucose measures were acted on in any way- were cut-offs used for action or diagnosis of GDM etc. When were these performed early or late? There was 1 person who had a HbA1c diagnostic of overt diabetes in pregnancy- presumably treated. Again because of lack of detailed analyses of these other tests of glycaemia- much less focus should be placed on these in the manuscript.

   Suggest- remove mention of HbA1c, fasting/random glucose from abstract.

For Figure 2 - reformat without HbA1c and fasting/random blood glucose.

Thank you for raising these important points. This issue was discussed extensively with the authorship group and other key stakeholders during the study. In our high-risk population, it was felt important to determine whether alternate tests such as an HbA1C or isolated plasma glucose levels were being used as alternatives when an OGTT was not performed. A key finding from our research is that in the vast majority of cases, there was still screening for overt diabetes (with an Hba1c) when an OGTT did not occur. Nevertheless, we have removed mention of HbA1C and glucose from the abstract as suggested. Whether or not the use of Hba1C is a valid approach and how it relates to GDM screening and outcomes will be explored further in a subsequent paper. This has been added to the discussion with the following sentences added to the discussion:

“Further research is needed to clarify the role of HbA1c testing in early pregnancy, including appropriate diagnostic thresholds. Data on the sensitivity and specificity of HbA1c in this context remain limited. The validity of HbA1c as a screening approach, and its relationship to gestational diabetes screening and pregnancy outcomes, will be examined in a subsequent analysis”.

In terms of whether an HbA1C result was acted on, local guidelines at time suggested that if an HbA1c was 5.7% and above and an OGTT not able to be performed, then they should be treated as having GDM. This with be further explored in the subsequent paper.

2. **Page 7- 2nd and 3rd paragraphs. As well as reporting the % of the whole cohort having hyperglycemia in pregnancy (GDM and ODIP) - can the % of those tested with an OGTT early and late also be included.**

   Thank you, these 2 paragraphs have been changed (with the addition of the bolded sentences) to incorporate this suggestion

“Hyperglycaemia in pregnancy was newly detected in 3.3% (n/N = 37/1115) of all pregnancies at early screening, with 2.8% (n/N = 31/1115) meeting criteria for GDM and 0.5% (n/N = 6/1115) meeting criteria for overt DIP. **When an early OGTT was performed, 16% (n/N = 37/226) met criteria for hyperglycaemia in pregnancy.** Of those who had a negative early OGTT, 36% (n/N = 68/189) did not go on to complete a routine OGTT later in pregnancy as per guidelines. An early HbA1c was performed in 69% (n/N = 774/1115) of all pregnancies without pre-existing diabetes. One woman had an early HbA1c ≥ 6.5%.

A routine OGTT was undertaken in 63% (n/N = 682/1078) of eligible pregnancies. Hyperglycaemia in pregnancy was detected in 11% (n/N = 117/1078), with 9% (n/N = 98/1078) meeting criteria for GDM and 1.3% (n/N = 14/1078) meeting criteria for overt DIP. **When a routine OGTT was performed, 17% (n/N = 117/682) met criteria for hyperglycaemia in pregnancy.**

3. **The subgroup analysis is helpful. Mention in the results section that the group that underwent early OGTTs were older with higher BMIs needs to be included.**

The following has been added to the results section, page 8:

“In this subgroup analysis, women who were screened with an early OGTT, compared to women did not have an early OGTT, had a higher BMI (27kg/m^2 vs. 24, p<0.01) and first presented for antenatal care earlier in gestation (8 weeks vs. 11, p<0.01) but were of similar age (27 years vs. 26, p =0.09).”

<u>**Reviewer 2**</u>

1. In my version of the manuscript (with track changes) there was a reference missing in the Introduction, page 4 (after, "more broadly in the literature").

Apologies, this has been corrected.

2. I would suggest, based on the author’s response to both reviewers’ comments regarding HbA1c as a screening method, adding these considerations in a bit more detail into the discussion. For example, a sentence explaining that there are not adequate sensitivity/specificity data currently available for the context, and that the research group is working on a follow-up manuscript to contribute to this lack of knowledge (just a bit more specific than the current sentence in the conclusion, which currently reads: "Rates of pregnancy HbA1c screening were higher, likely reflecting greater acceptability and convenience of this test. Future research should further evaluate the role of HbA1c testing in early pregnancy in this context, including consideration of diagnostic thresholds.").

Thank you for this important comment. The following has been added to the discussion on page 11:

“Further research is needed to clarify the role of HbA1c testing in early pregnancy, including appropriate diagnostic thresholds. Data on the sensitivity and specificity of HbA1c in this context remain limited. The validity of HbA1c as a screening approach, and its relationship to gestational diabetes screening and pregnancy outcomes, will be examined in a subsequent analysis.”
